# Modulatory Effects of the Piccolo Genotype on Emotional Memory in Health and Depression

**DOI:** 10.1371/journal.pone.0061494

**Published:** 2013-04-19

**Authors:** Saskia Woudstra, Marie-José van Tol, Zoltán Bochdanovits, Nic J. van der Wee, Frans G. Zitman, Mark A. van Buchem, Esther M. Opmeer, André Aleman, Brenda W. Penninx, Dick J. Veltman, Witte J. Hoogendijk

**Affiliations:** 1 Department of Psychiatry, VU University Medical Center, Amsterdam, The Netherlands; 2 Department of Medical Genomics, VU University Medical Center, Amsterdam, The Netherlands; 3 Department of Psychiatry, Leiden University Medical Center, Leiden, The Netherlands; 4 Leiden Institute for Brain and Cognition, Leiden University, Leiden, The Netherlands; 5 Neuroscience Campus Amsterdam, VU University, Amsterdam, The Netherlands; 6 NeuroImaging Center & Department of Neuroscience, University Medical Center Groningen, University of Groningen, Groningen, The Netherlands; 7 Clinical Affective Neuroimaging Laboratory, Center for Behavioral Brain Sciences, Otto-von-Guericke University, Magdeburg, Germany; 8 Department of Behavioral Neurology, Leibniz Institute for Neurobiology, Magdeburg, Germany; 9 Department of Radiology, Leiden University Medical Center, Leiden, The Netherlands; 10 Department of Psychology, Groningen University, Groningen, The Netherlands; 11 Department of Psychiatry, Erasmus Medical Center, Rotterdam, The Netherlands; University of Wuerzburg, Germany

## Abstract

Major depressive disorder (MDD) has been associated with biased memory formation for mood-congruent information, which may be related to altered monoamine levels. The piccolo (*PCLO*) gene, involved in monoaminergic neurotransmission, has previously been linked to depression in a genome-wide association study. Here, we investigated the role of the *PCLO* risk allele on functional magnetic resonance imaging (MRI) correlates of emotional memory in a sample of 89 MDD patients (64 *PCLO* risk allele carriers) and 29 healthy controls (18 *PCLO* risk allele carriers). During negative word encoding, risk allele carriers showed significant lower activity relative to non-risk allele carriers in the insula, and trend-wise in the anterior cingulate cortex and inferior frontal gyrus. Moreover, depressed risk allele carriers showed significant lower activity relative to non-risk allele carriers in the striatum, an effect which was absent in healthy controls. Finally, amygdalar response during processing new positive words vs. known words was blunted in healthy PCLO+ carriers and in MDD patients irrespective of genotype, which may indicate that signalling of salient novel information does not occur to the same extent in PCLO+ carriers and MDD patients. The *PCLO* risk allele may increase vulnerability for MDD by modulating local brain function with regard to responsiveness to salient stimuli (i.e. insula) and processing novel negative information. Also, depression-specific effects of PCLO on dorsal striatal activation during negative word encoding and the absence of amygdalar salience signalling for novel positive information further suggest a role of PCLO in symptom maintenance in MDD.

## Introduction

Major depressive disorder (MDD) is a highly prevalent psychiatric disorder, with twin studies showing that up to 40% of MDD is genetically determined [1]. Phenotypically, depression is characterized by depressed mood and/or anhedonia (loss of interest in nearly all activities) and has been associated with attentional deficits, resulting in poor functioning in daily life [2], [3]. Symptoms of negative mood, lack of positive affect, and attentional impairments may ensue from, or be reinforced by, dysfunctional emotional memory processes. Phenotypic features of abnormal perception, encoding, and consolidation of emotional information, often seen in depression, may be moderated by altered monoamine levels. Much of the candidate gene literature has focused on genes from the monoaminergic neurotransmitter system, such as the serotonin transporter, monoamine oxidase A and tryptophan hydroxylase 2 [4]–[9]. A recent study by our consortium demonstrated that candidate genetic association studies are not well replicated [10], which suggests that a hypothesis-free approach is more useful to identify possible genetic variants that contribute to MDD. A genome-wide association study for MDD found the SNP rs2522833 located at position 82453708 (hapmap genome build 37.1) in the piccolo gene (*PCLO*), which is involved in monoaminergic neurotransmission, to be of particular interest in its genetic model [11]. This association was confirmed in a number of studies with similar and related phenotypes [12]–[17], but not in others [18]–[20]. The rs2522833 SNP alters the hydrophilic, uncharged aminoacid serine to the charged aminoacid alanine in the calcium-binding C2A domain of PCLO and may affect protein stability [21]. The PCLO protein is localized at the cytomatrix of the presynaptic active zone and is important in monoaminergic neurotransmission in the brain [13], [20]. Recently, we have shown that the risk allele on the piccolo gene in healthy controls and depressed patients was associated with abnormal processing of negative emotional faces rather than executive functioning [12]. However, whether the rs2522833 polymorphism in the *PCLO* gene can also affect emotional memory processing has not been studied yet.

On a cognitive-behavioural level, MDD has been associated with attentional bias towards mood-congruent (i.e. negative) information [22]. Using neuropsychological assessments [23], it has been shown that negative emotional processing bias may be predictive of depression symptoms and may represent a state marker of MDD. It is thought that this negative bias is associated with abnormal responsiveness of brain regions involved in emotion processing, as well as disruption of cortico-limbic connections that are important for regulating emotional responses [24]. Recently we found an association of memory processing of positive and negative information in MDD with altered activity in the amygdala, ventral striatum, insula, hippocampus, anterior cingulate cortex (ACC), and inferior frontal gyrus (IFG) [25]. We found left ventral insular activation specifically during processing of negative words [25], which may reflect a general increased sensitivity for negative information, as suggested by Surguladze *et al*. (2010) [26]. In another study, an abnormal response for recollecting negative faces was found in MDD patients, which may reflect activation of negative schemas [27]. MDD has also been associated with a mood-incongruent bias (i.e., away from positive information) [28]–[30] which may affect memory formation for positive as compared to negative and neutral stimuli. Using event related potentials (ERP), Shestyuk *et al*. observed smaller slow wave amplitudes to positive self-relevant words in MDD relative to controls, whereas group differences for negative or neutral stimuli were absent [30]. In summary, negative and positive biases may lead to abnormal memory formation, reinforcing negative mood and further contributing to a chronic course of the disorder [31], [32].

Recent studies have underscored the importance of PCLO in MDD [13]–[16], and in neural processes underlying memory formation [33]. Although we recently found evidence for the *PCLO* risk allele to be associated with emotional processing of negative faces, it remains unclear whether effortful classification of emotional words is characterized by a similar association. Moreover, it is unknown whether PCLO modulates negative bias and emotional memory in depression.

Over the last few years, imaging genetics has shown to be a powerful method for investigating neurobiological pathways in various psychiatric disorders [34], [35]. Using an intermediate phenotype, such as emotional memory processing, which is probably closer to the neurobiological substrate of MDD than the clinical diagnosis itself [36], may be helpful in identifying genetic risk alleles. Until now, imaging genetics studies on emotional memory have mainly been conducted in healthy controls and in psychiatric disorders other than MDD. We have recently studied emotional processing and executive function in the context of genetic association with *PCLO* in a group of MDD patients and healthy controls [12] and found an association increased amygdalar activity during the processing of negative faces. Considering that encoding and retrieval of emotional stimuli is a complex form of cognitive and emotional processing, we hypothesize that the *PCLO* risk allele will also modulate emotional, especially negative, memory processing. Focussing on the amygdala, ventral striatum, hippocampus, anterior cingulate cortex (ACC), insula, and inferior frontal gyrus (IFG), regions that are important for encoding and recognition of valenced semantic information, we studied functional MRI correlates of successful emotional word encoding and recognition, and focussed on activation patterns explained by *PCLO* genotype in these areas independent of psychopathology. Since the pathophysiology of MDD is complex and diverse, we also investigated whether *PCLO* genotype effects on the brain were different in the presence of MDD psychopathology. In addition, we controlled for the use of selective serotonin reuptake inhibitors (SSRI) and tested whether functional effects coincided with morphometric variations related to *PCLO* genotype.

## Methods

### Participants

The present study was part of a large imaging study (details described elsewhere [25]) included in the Netherlands Study of Depression and Anxiety (NESDA) [37]. After excluding participants due to missing *PCLO* genotype data, technical problems during scanning and/or insufficient task performance, our final sample consisted of 89 MDD patients and 29 healthy controls (see figure S1 in File S1 for a detailed flowchart of the numbers of participants included). Exclusion criteria were: presence of MRI contraindications, DSM-IV axis I disorder other than MDD, Panic Disorder (PD) or Social Anxiety Disorder (SAD) (except Generalized Anxiety Disorder/GAD) lifetime, or any DSM-IV disorder (for HC), dependence or recent abuse of alcohol and/or drugs, hypertension, major internal and/or neurological disorders, and use of psychotropic medication (other than stable use of a selective serotonin reuptake inhibitor [SSRI] or incidental use of benzodiazepines). To assess depressive symptom characteristics and severity scores, the Inventory of Depressive Symptomatology (IDS) [38], and the Montgomery-Åsberg Depression Rating Scale (MADRS) [39], were used. PCLO groups did not differ with regard to age, gender, education, MDD/HC ratio, depression severity, or SSRI use/duration (see Table 1). All participants provided written informed consent and the Ethics Committees at the VU University Medical Center, and Academic Medical Center, Amsterdam, the Leiden University Medical Center and at the University Medical Center Groningen approved this study.

**Table 1:** Sample characteristics and task performance.

		Group; mean (SD)			
	total *(n = 118)*	PCLO+ *(n = 82)*	PCLO− *(n = 36)*	p-value	?^2^
**Sample characteristics**					
Gender (%female)	61.9 (n = 73)	62.2 (n = 51)	61.1 (n = 22)	1	.012
Age (years)	38.1 (10.2)	37.5 (10.1)	39.3 (10.6)	.42	
Education (years)	12.6 (3.2)	12.9 (3.3)	12.0 (2.8)	.16	
Scancenter (% A, L, G)	28.8, 41.5, 29.7	26.8, 43.9, 29.3	33.3, 36.1, 30.6	.69	.747
Diagnosis (MDD/HC)	89/29	64/18	25/11	.36	.999
IDS (score)	18.8 (12.9)	19.7 (12.4)	16.9 (14.1)	.28	
MADRS (score)	12.5 (10.5)	13.4 (10.4)	10.3 (10.5)	.14	
Duration SSRI use (months)	18.9 (28.8)	14.5 (19.8)	32.0 (46.6)	.17	
SSRI use (no/yes)	90/28	61/21	29/7	.64	.525
**Memory performance (p)**					
CREC.neg	.685 (.138)	.685 (.139)	.685 (.135)	.99	
CREC.pos	.725 (.132)	.719 (.132)	.739 (.132)	.45	
CREC.neu	.69 (.166)	.692 (.155)	.688 (.191)	.90	
CREC.all	.70 (.122)	.697 (.122)	.70 (.124)	.83	
FA.neg	.17 (.11)	.17 (.10)	.18 (.11)	.72	
FA.pos	.12 (.11)	.13 (.11)	.11 (.11)	.35	
FA.neu	.07 (.06)	.07 (.07)	.06 (.06)	.72	
FA.all	.124 (.079)	.125 (.08)	.119 (.079)	.71	
CREJ.neg	.66 (.141)	.66 (.137)	.67 (.151)	.623	
CREJ.pos	.70 (.175)	.69 (.175)	.72 (.175)	.503	
CREJ.neu	.81 (.13)	.81 (.13)	.82 (.131)	.699	
CREJ.all	.729 (.13)	.72 (.127)	.74 (.137)	.54	
**Response time (sec)**					
rt SCR.neg	1.296 (.385)	1.311 (.411)	1.263 (.321)	.63	
rt SCR.pos	1.487 (.379)	1.507 (.384)	1.441 (.368)	.45	
rt SCR.neu	1.563 (.393)	1.567 (.415)	1.553 (.343)	.69	
rt CREC.neg	1.264 (.256)	1.265 (.237)	1.263 (.30)	.61	
rt CREC.pos	1.355 (.289)	1.37 (.291)	1.319 (.287)	.27	
rt CREC.neu	1.339 (.297)	1.335 (.276)	1.349 (.346)	.90	
rt FA.neg	1.50 (.45)	1.48 (.42)	1.53 (.50)	.65	
rt FA.pos	1.62 (.52)	1.59 (.41)	1.69 (.71)	.37	
rt FA.neu	1.58 (.53)	1.60 (.51)	1.53 (.57)	.56	
rt CREJ.neg	1.48 (.35)	1.50 (.33)	1.45 (.39)	.52	
rt CREJ.pos	1.52 (.34)	1.54 (.34)	1.50 (.35)	.53	
rt CREJ.neu	1.39 (.31)	1.40 (.31)	1.37 (.33)	.61	

Sample characteristics and task performance of the total sample. SD: standard deviation; n:number of participants; PCLO+: *PCLO* risk allele carriers; PCLO−: *PCLO* non-risk allele carries; A: Amsterdam; L: Leiden; G:Groningen; MDD: major depressive disorder; HC: healthy controls; IDS: Inventory of Depressive Symptomatology; MADRS: Montgomery-Åsberg Depression Rating Scale; SSRI: Selective Serotonin Reuptake Inhibitors; p: proportion correct answers; neg: negative (words); pos: positive (words); neu: neutral (words); sec: seconds; rt: response time.

Mean proportion correct and response times for encoding (subsequent hits) and recognition (hits, false alarms, correct rejection) indices. Correct RECognition (CREC): correct recognition of a previously encoded word; False Alarm (FA): incorrect indication of a newly presented word as a previously encoded word; Correct REJection (CREJ): correct recognition of a newly presented word as a new word; Subsequent Correct Recognition (SCR): consists of a word, presented during the encoding phase, that is correctly recognized during the subsequent recognition phase.

### Genotyping

As described in detail elsewhere [11], genotyping was performed by Perlegen. Observed genotypes in our sample did not deviate from Hardy-Weinberg equilibrium (CC:AC:AA = 25∶57:36; χ^2^[1] = 0.08; *p*>0.05). All subjects reported Western European ancestry. We formed two groups based on the *PCLO* genetic association study in MDD. One group consisted of participants carrying the risk allele (AC/CC), and one group included participants not carrying the risk allele (AA). In the following, we will refer to these groups as PCLO+ (risk allele) carriers and PCLO− carriers, respectively.

### Emotional Memory Task Paradigm

An event-related, (subject-paced), word encoding and recognition paradigm was used which has been described extensively elsewhere [25]. During the encoding part, participants were asked to classify 40 positive, 40 negative, and 40 neutral words according to their valence. Words were presented pseudo-randomized together with 40 baseline trials in 20 blocks of eight words. After a brief retention interval, participants were asked to complete a word recognition task. This task consisted of the 120 old encoding target words and 120 new distracter words (matched for valence), and 40 baseline trials, presented pseudo-randomized in 20 blocks of 14 words. Participants had to indicate whether they had ‘seen’ (i.e. remembered) the words previously, ‘probably seen’ (‘know’), or ‘not seen’ (rejection).

### Magnetic Resonance Imaging Data Acquisition

The functional neuroimaging methods have been comprehensively reported elsewhere [40], [41]. In summary, T2*-weighted echo-planar images (EPI) sensitive to the blood oxygenation level–dependent (BOLD) effect were acquired using similar Philips 3T MR systems (repetition time [TR] = 2300 ms, echo time [TE] = 30.0 ms (UMCG: 28.0 ms), 35 slices (UMCG 39 slices)), situated at different locations (Amsterdam, Leiden, and Groningen, the Netherlands).

The EPI volumes were acquired at 35 slices (UMCG: 39 slices), interleaved axial acquisition, 3 mm thickness, matrix size 96×96 (UMCG: 64×64), in-plane resolution 2.29×2.29 mm (UMCG: 3×3 mm). A T1-weighted anatomical MRI was also acquired for each subject and included a sagittal 3-dimensional gradient-echo sequence (TR = 9 ms, TE = 3.5 ms, matrix 256×256, voxel size: 1×1×1 mm, 170 slices).

### Statistical Analysis

#### Performance

Responses and response times were recorded and were used to calculate proportions (p) Hits, correct rejections (pCREJ), False Alarms (pFA), and old/new discriminant accuracy (d’ = pHits-pFA), overall and per valence (negative, neutral, and positive). Repeated-measures analyses of covariance (ANCOVAs) were performed to test for effects of *PCLO* genotype, genotype × diagnosis effects, and interaction effects of genotype, diagnosis and genotype × diagnosis with valence on task performance (pHits_all, pFalseAlarms_all, and d’_all) and response times during successful encoding and successful recognition. Significance for behavioural analyses was set at *P*<.05 and post hoc paired tests (T-test or Mann-Whitney [U] were Bonferroni-corrected for multiple comparisons (*P*_Bonferroni_).

#### Imaging data analysis

Image processing was performed using Statistical Parametric Mapping (SPM5; http://www.fil.ion.ucl.ac.uk/spm/software/spm5; software implemented in Matlab 7.5.0 (The Matlab Inc, Natick, MA, USA). Details of preprocessing and first-level single-subject analyses have been described elsewhere [25]. Briefly, following temporal and spatial preprocessing (final smoothing: 8 mm full-width at half-maximum [FWHM]), data were analyzed in the context of the General Linear Model. The subject-specific first-level models included regressors for encoding and recognition events. Due to the small proportion of recognition trials that were responded to with a ‘know’ response, these responses were treated as ‘remembered’ and consequently added to either correct recognized (CREC) or false alarms (FA). Activation maps associated with different valences were calculated per subject. To avoid inclusion of non-task related signal which might be expected when contrasting against the repetitive lower baseline, contrast images with visual input that only differed in its emotional content (‘encoding positive > encoding neutral’, ‘encoding negative > encoding neutral’ resulting from the encoding phase, and ‘recognized positive > recognized neutral’, and ‘recognized negative > recognized neutral’ were likewise included in a second-level random-effect analysis. Although our primary aim was to investigate valence effects on processes of word encoding and recognition, we also investigated specific effects of successful recognition by setting up the following contrast: ‘correct recognition (hits) > correct rejection’, resulting from the recognition phase) and included this contrast in a second-level random-effect analysis.

Based on our previous study [25] we included the following regions of interest for the emotional memory task, defined using the Automated Anatomical Labeling (AAL) atlas or Talairach Daemon (for the striatum) [42], implemented in the Wake Forest University (WFU) Pick Atlas toolbox: amygdala, hippocampus, ACC, IFG, insula, and striatum (includes caudate head, tail, body, putamen). Main effects of task are reported at a threshold of *P*<.05, whole-brain corrected for False Discovery Rate (FDR). We conducted a full factorial using genotype and diagnosis as between subject factors, and valence per encoding or recognition as within-subject factor, to test whether the PCLO+ was associated with altered activity in our ROIs for each valence for encoding or recognition. Scan location was entered as covariate by means of two dummy variables. Furthermore, to test for the specificity of valence for correctly recognized old versus new words, we conducted a 2×2×3 ANOVA with genotype (PCLO+, PCLO−) and diagnosis (MDD, HC) as between-subject factor, and valence (positive, negative, and neutral; e.g. CREC_positive > CREJ_positive) as within-subject factor. Scan location was entered as covariate by means of two dummy variables. Main effects of genotype and interaction of *PCLO* genotype with current psychopathology were reported at a voxel-wise threshold of *P*<.05 FDR corrected for the regions of interest, with an initial threshold of *P*<.001 uncorrected. In addition, to explore activity common to both negative and positive vs. neutral stimuli we post hoc computed the conjunction of these two contrasts, based on the global null hypothesis (k≥1) [43]. Each contrast had to meet a threshold of *P*<.001 uncorrected. To test whether between-group effects were related to volumetric differences, we conducted a two-by-two ANOVA for those regions that showed a between-group effect during the functional paradigm. Effects were reported at a threshold of *P*<.05 FDR corrected for the region of interest. To account for the number of *a priori* regions of interest, we corrected the critical corrected *p*-value for the number of regions (n = 6: amygdala, hippocampus, ACC, IFG, insula, and striatum). Using a standard Bonferroni correction would be too stringent, however, since the dependent variables were measured within the same individuals. Therefore, we took this interdependency into account and calculated the optimal threshold for positive vs. neutral and negative vs. neutral encoding and recognition using the Simple Interactive Statistical Analysis Bonferroni tool (www.quantitativeskills.com/sisa/calculations/bonfer.htm). Response in the six *a priori* regions of interest showed a mean correlation of r = .75 during positive encoding (>neutral encoding) across all participants, r = .63 (>neutral encoding) during negative encoding, r = .84 (>neutral recognition) during positive recognition, and r = .69 (>neutral recognition) during negative recognition, leading to a critical *alpha* of.026,.032,.037, and.029, respectively (Table S2 in File S1). Because SSRI use may alter regional brain function in psychiatric diseases [44], we repeated our analyses omitting SSRI users, to test for possible effect of SSRI use.

## Results

### Sample Descriptives

Table 1 lists the sample characteristics and behavioural statistics. Genotype groups were matched for MDD diagnosis, age, gender, and education.

Behavioural analyses of the emotional word encoding and recognition task revealed no significant main effect of genotype, no genotype × valence, and no genotype × valence × diagnosis interaction on accuracy and response time indices.

Table 2 lists the genotype × diagnosis sample and behavioural characteristics.

**Table 2:** Sample characteristics and task performance group × diagnosis.

	Group; mean (SD)			
	PCLO+		PCLO−	
	MDD (N = 64)	HC (N = 18)	MDD (N = 25)	HC (N = 11)
**Sample characteristics**				
Gender (%female)	65.6% (n = 42)	50.0% (n = 9)	60% (n = 15)	63.6% (n = 7)
Age (years)	35.9 (9.57)	43.2 (10.05)	39.0 (11.38)	40.0 (9.12)
Education (years)	12.4 (3.24)	14.7 (2.78)	11.3 (2.85)	13.6 (2.16)
Scancenter (% A, L, G)	23.4, 46.9, 29.7	38.9, 33.3, 27.8	24, 36, 40	54.5, 36.4, 9.1
IDS (score)	24.3 (10.03)	3.9 (3.26)	22.1 (13.29)	3.7 (3.43)
MADRS (score)	16.8 (9.12)	1.5 (2.18)	14.4 (9.82)	.27 (.65)
Duration SSRI use (months)	14.5 (19.76)	N/A	32.0 (46.59)	N/A
SSRI use (no/yes)	43/21	18/0	18/7	11/0
**Memory performance (p)**				
pCREC.neg	.693 (.143)	.658 (.127)	.700 (.118)	.652 (.170)
pCREC.pos	.724 (.129)	.700 (.145)	.728 (.143)	.764 (.103)
pCREC.neu	.702 (.163)	.656 (.114)	.717 (.175)	.621 (.219)
pFA.all	.123 (.084)	.132 (.067)	.129 (.078)	.099 (.079)
pCREJ.neg	.658 (.136)	.668 (.143)	.655 (.146)	.718 (.161)
pCREJ.pos	.696 (.165)	.701 (.212)	.713 (.165)	.739 (.205)
pCREJ.neu	.807 (.136)	.846 (.106)	.800 (.142)	.884 (.083)
**Response time (sec)**				
rt SCR.neg	1.28 (.32)	1.32 (.57)	1.26 (.26)	1.24 (.37)
rt SCR.pos	1.53 (.39)	1.35 (.31)	1.43 (.36)	1.45 (.26)
rt SCR.neu	1.59 (.39)	1.47 (.45)	1.52 (.32)	1.54 (.31)
rt CREJ.neg	1.51 (.32)	1.48 (.35)	1.44 (.42)	1.50 (.35)
rt CREJ.pos	1.56 (.34)	1.49 (.34)	1.50 (.37)	1.50 (.33)
rt CREJ.neu	1.41 (.31)	1.40 (.30)	1.37 (.37)	1.37 (.24)
rt CREC.neg	1.26 (.21)	1.33 (.30)	1.23 (.30)	1.29 (.25)
rt CREC.pos	1.36 (.27)	1.42 (.31)	1.31 (.29)	1.30 (.24)
rt CREC.neu	1.31 (.25)	1.43 (.30)	1.31 (.31)	1.40 (.35)

Sample characteristics and task performance of the total sample, divided into genotype × diagnosis. SD: standard deviation; n:number of participants; PCLO+: *PCLO* risk allele carriers; PCLO−: *PCLO* non-risk allele carries; A: Amsterdam; L: Leiden; G:Groningen; MDD: major depressive disorder; HC: healthy controls; IDS: Inventory of Depressive Symptomatology; MADRS: Montgomery-Åsberg Depression Rating Scale; SSRI: Selective Serotonin Reuptake Inhibitors; p: proportion correct answers; neg: negative (words); pos: positive (words); neu: neutral (words); sec: seconds; rt: response time.

Mean proportion correct and response times for encoding (subsequent hits) and recognition (hits, false alarms, correct rejection) indices. Correct RECognition (CREC): correct recognition of a previously encoded word; False Alarm (FA): incorrect indication of a newly presented word as a previously encoded word; Correct REJection (CREJ): correct recognition of a newly presented word as a new word; Subsequent Correct Recognition (SCR): consists of a word, presented during the encoding phase, that is correctly recognized during the subsequent recognition phase.

### Imaging Results

Main effects of the encoding and recognition contrast across groups can be found in Table 3. Table S3 in File S1 describes the main effects of encoding and recognition per valence.

**Table 3:** Main effects of encoding of emotional words vs. neutral words.

Emotional Encoding								
	Side	BA	MNI coordinates				p (FDR)	k^a^
Regions			x	y	z	Z		
*Frontal*								
Inferior Frontal Gyrus	R	45	54	24	6	3.82	0.004	68
	R	47	42	21	−15	3.41	0.009	10
Medial Frontal Gyrus	L	10	−3	57	−3	5.2	0.004	513
	L	10	−3	60	21	4.72	0.001	513
	R	6	3	48	39	3.16	0.014	455
Middle Frontal Gyrus	L	10	−33	42	30	2.83	0.025	11
	R	6	48	3	48	3.82	0.004	317
	R	6	36	−6	57	3.86	0.004	317
	L	9	−33	33	39	2.71	0.031	11
*Temporal/Parietal*								
Precentral Gyrus	L	6	−30	−9	54	5.29	0.001	4504
Anterior Cingulate	L	32	−6	27	30	3.61	0.006	226
	R	24	6	18	33	3.31	0.01	226
Posterior Cingulate	R	30	30	−72	9	3.68	0.005	1574
*Subcortical*								
Amygdala	L	N/A	−24	3	−24	2.74	0.032	10
Putamen	L	N/A	−21	9	−3	3.04	0.023	15
Cuneus	L	30	−15	−72	9	3.56	0.011	394
Lingual Gyrus	L	18	−12	−75	−9	3.71	0.011	394
Inferior Parietal Lobule	R	40	45	−33	42	4.29	0.002	1574
Medial Dorsal Nucleus	L	thalamus	−3	−15	9	4.04	0.003	265
Middle Temporal Gyrus	L	21	−51	3	−24	5.4	0.001	4504
Superior Temporal Gyrus	R	38	48	6	−27	3.25	0.012	19
Supramarginal Gyrus	R	40	63	−48	24	3.93	0.003	1574

Main effects of encoding of emotional words vs. neutral words. Main effects are reported at *P*_FDR_<.05, whole brain corrected with a minimum cluster size of 10. MNI: Montreal Neurological Institute; BA: Brodmann area; k: clustersize; L: left; R: right; a: clustersize at *p*<.05 FDR whole brain corrected.

**Figure 1:**
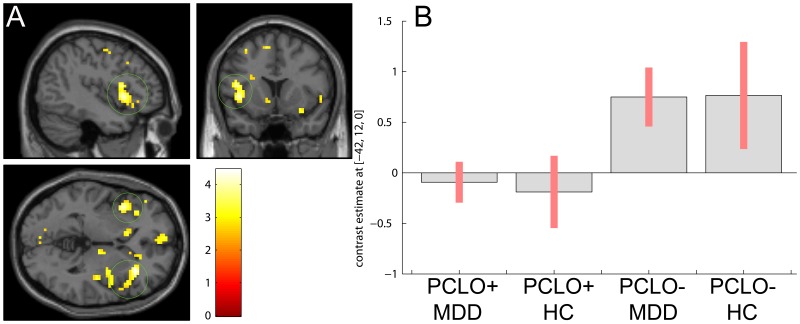
PCLO genotype effect during negative word encoding in the insula. PCLO+ carriers show significant hypoactivation of the insula, relative to PCLO− carriers during negative word encoding. Panel A shows sagittal, coronal, and axial section at peak activation (green circle: insula; results shown at *P*<.005; Z = 3.92 (left), Z = 4.26 (right)). Panel B shows the cluster means for each peak voxel, with their standard errors for the different groups. PCLO: Piccolo genotype; PCLO+: PCLO risk allele carriers; PCLO−: PCLO non-risk allele carriers; MDD: Major Depressive Disorder; HC: Healthy controls; AU: arbitrary units.

**Figure 2:**
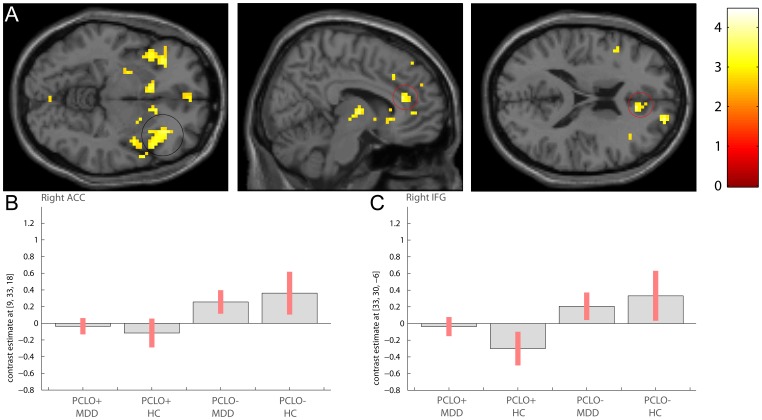
PCLO genotype effect during negative word encoding in the inferior frontal gyrus and anterior cingulate cortex. PCLO+ carriers show trend-wise hypoactivation of the inferior frontal gyrus and anterior cingulate cortex relative to PCLO− carriers during negative word encoding. Panel A shows sagittal, coronal, and axial section at peak activation (black circle: IFG, red circle: dorsal part of pgACC; results shown at *P*<.005; IFG, Z = 3.79 (left), Z = 3.42 (right); pgACC, Z = 3.12 (left), Z = 3.49 (right)). Panel B shows the cluster means for each peak voxel, with their standard errors for the different groups. PCLO: Piccolo genotype; PCLO+: PCLO risk allele carriers; PCLO−: PCLO non-risk allele carriers; MDD: Major Depressive Disorder; HC: Healthy controls; AU: arbitrary units.

**Figure 3:**
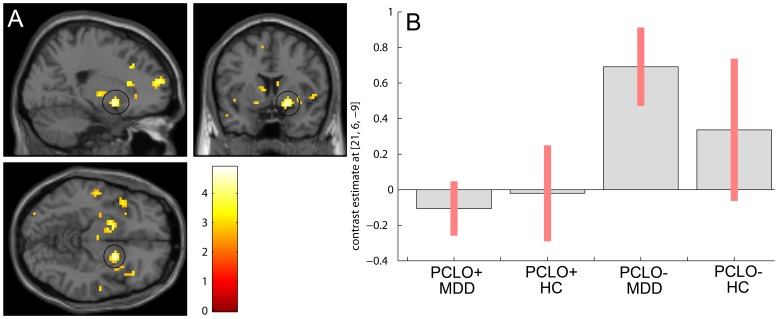
Genotype × diagnosis interaction during negative word encoding in the striatum. PCLO+ carriers within the MDD group show reduced striatal activity relative to the PCLO− carriers within the MDD group during negative word encoding. No effect of genotype is seen in the HC group. Panel A shows sagittal, coronal, and axial section at peak activation (results shown at *P*<.005; black circle: dorsal putamen, Z = 4.65 (right). Panel B shows the cluster means for each peak voxel, with their standard errors for the different groups. PCLO: Piccolo genotype; PCLO+: PCLO risk allele carriers; PCLO−: PCLO non-risk allele carriers; MDD: Major Depressive Disorder; HC: Healthy controls; AU: arbitrary units.

A significant main effect of genotype was observed during successful negative word encoding (‘encoding negative > encoding neutral’) in the insular cortex (left insula; MNI [−42 12 0]; Z = 3.92; right insula; *P*_FDR_ = .008 corrected for small volume; MNI [33 27 −3]; Z = 4.26; *P*_FDR_ = .007 corrected for small volume; Figure 1; Table S4 in File S1) and trend-wise in the dorsal part of the pregenual ACC and inferior frontal gyrus (left pregenual ACC; MNI [−3 33 30]; Z = 3.12; *P*_FDR_ = .046 corrected for small volume; right pregenual ACC; MNI [9 33 18]; Z = 3.49; *P*_FDR_ = .046 corrected for small volume; left IFG; MNI [−36 30 −3]; Z = 3.41; *P*_FDR_ = .045 corrected for small volume; right IFG; MNI [33 30 −6]; Z = 3.42; *P*_FDR_ = .045 corrected for small volume; Figure 2; Table S4 in File S1). This was due to lower activity in PCLO+ compared to PCLO−, which was observed independent of diagnostic status. An interaction of *PCLO* genotype and diagnosis was observed in the striatum (left ventral striatum (caudate head); MNI [−18 21 −3]; Z = 3.6; *P*_FDR_ = .028 corrected for small volume; (caudate body); MNI [−9 3 9]; Z = 3.3; *P*_FDR_ = .028 corrected for small volume; right ventral striatum (caudate body); MNI [9 0 15]; Z = 3.22; *P*_FDR_ = .028 corrected for small volume; left dorsal putamen; MNI [−18 3 −9]; Z = 3.57; *P*_FDR_ = .028 corrected for small volume; right dorsal putamen; MNI [21 6 −9]; Z = 4.65; *P*_FDR_ = .002 corrected for small volume. In the MDD group we found reduced activity in the PCLO+ carriers relative to the PCLO− carriers in this region, which was absent in healthy controls (Figure 3).

Effects of genotype during successful recognition of negative words did not meet the required threshold of *P*<.05 FDR corrected. Subtreshold at *P*<.001 uncorrected, increased activity in the insula in PCLO+ carriers relative to PCLO− carriers was observed (Table S4 in File S1).

During encoding of positive words or during recognition of positive words effects of genotype likewise did not meet the required *a priori* threshold. Exploration of these contrasts at a threshold of *P*<.001 uncorrected revealed decreased activity in frontal and limbic areas for encoding positive words (regions are listed in table S4 in File S1).

During correct recognition>correct rejection (in the recognition phase) of positive words we found a significant interaction of emotion × diagnosis × genotype: During rejection of positive new words, healthy PCLO− carriers showed increased left amygdalar activation, while no difference between processing positive old and new words was observed in PCLO+ carriers and in MDD patients, indicating blunting to novel positive information in PCLO+ carriers and patients (MNI [−27 −3 −24]; Z = 3.36; Figure S5 in File S1). No effect of negative or neutral words was found.

**Figure 4:**
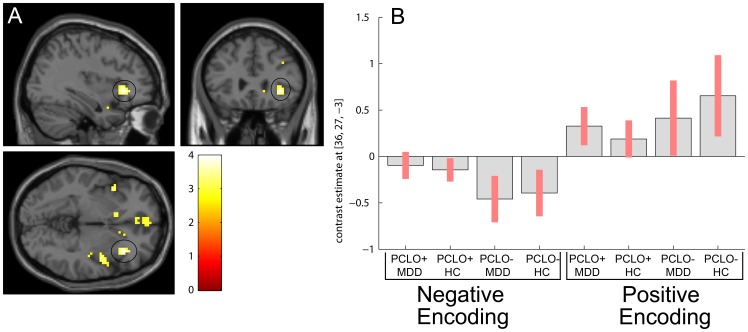
Valence specific effects during word encoding. Panel A shows valence specific effects during word encoding (results shown at *P*<.005). Black circle: inferior frontal gyrus (right); MNI [x = 36 y = 27 z = −3], Z = 3.9. Panel B: Parameter estimates and 95% confidence intervals showing direction of effect for each valence during word encoding in peakvoxel (MNI [x = 36 y = 27 z = −3]). PCLO: Piccolo genotype; PCLO+: PCLO risk allele carriers; PCLO−: PCLO non-risk allele carriers; MDD: Major Depressive Disorder; HC: Healthy controls; AU: arbitrary units.

We tested post hoc for common valence effects by performing a conjunction analysis, using both the negative and positive word encoding contrasts. We found a *PCLO* genotype effect in regions including IFG, medial frontal, insula, and caudate head (Figure 4), reflecting reduced activity of the PCLO+ carriers relative to PCLO− carriers. It should be noted that our significant conjunction (although at an explorative threshold of p<.001 uncorrected) does not mean all the contrasts were individually significant (i.e., a conjunction of significance). It indicates that the contrasts were consistently high and jointly significant. This is equivalent to inferring one or more effects were present.

No volumetric differences were observed between groups in these regions.

### Effects of SSRI

After excluding SSRI users (n = 28), PCLO effects observed for processing of emotional words memory (‘encoding negative vs. encoding neutral, encoding positive vs. encoding neutral, recognition negative vs. recognition neutral, and recognition positive vs. recognition neutral’) were similar to the main genotype analyses.

## Discussion

In the present study, we examined the effects of the *PCLO* rs2522833 polymorphism on regional brain activation during performance of an emotional word encoding and recognition paradigm in MDD patients and healthy controls, while also controlling for SSRI use and volumetric differences. Just below threshold, results indicated that PCLO is associated with psychopathology-independent functional changes within the dorsal part of the pregenual (pg)ACC, predominantly during processing of novel, negative information. Whereas pgACC hypoactivation in PCLO+ carriers was specific for the processing of negative information, we found that the *PCLO* risk allele modulated both negative and positive information processing in the IFG and insula. In addition, PCLO was found to differentially affect striatal activation during negative encoding in health and disease, as genotype effects were observed in MDD patients but not in controls. Successful recognition of emotional words was not associated with significant PCLO effects. To avoid problematic interpretation of genotype effects on memory processing [45], [46], memory performance was modelled at first level. Moreover, we found a blunted effect in the amygdala in PCLO+ carriers and MDD patients of new positive words relative to old words, which may indicate that signalling of salient novel information does not occur to the same extent in PCLO+ carriers and MDD patients. During recognition of negative or neutral words, no difference between PCLO+ and PCLO− was found. We found no effect of *PCLO* genotype, or PCLO × MDD interaction during performance.

To our knowledge, this is the first genetic neuroimaging association study in MDD and healthy controls that shows an effect of *PCLO* genotype related hypoactivation of insula, and trend-wise of ACC and IFG during emotional memory processing. In a previous study, we showed that the *PCLO* risk allele is associated with abnormal involvement of limbic regions in response to negative stimuli (i.e. emotional faces), but not with altered prefrontal recruitment during an executive control task [12]. In the present study, we extended these findings by showing that in PCLO+ carriers, processing of negative information (words) is characterized by reduced insula, and trend-wise pgACC and IFG activation. These regions are considered important regions in the production and effortful regulation of mood states, and have been consistently associated with the psychopathology of MDD [47]. Moreover, the insula has been implicated in the salience network [48], where it is considered an important hub for processing salient events for action to be initiated, including calling on attentional resources and regulating autonomic activity in reaction to salient stimuli [49]. Although near-significant only, the present results demonstrate that PCLO impacts on processing of negative information in the dorsal part of the pgACC in both healthy controls and depressed patients. Our finding that *PCLO* genotype modulates the processing of negative novel information specifically in the pgACC replicates results obtained in healthy controls [50]–[52]. Therefore, these findings indicate that biases towards negative stimuli, as reflected in altered pgACC activation, may represent not only a feature of MDD, but also a vulnerability factor for developing mood disorders [53].

Taken together, this suggests that *PCLO* genotype may increase the risk for developing or maintaining a depressive disorder by affecting the mood regulating capacity of the brain.

In contrast to these psychopathology-independent findings, decreased activity in the ventral striatum during processing of negative stimuli was found in MDD patients only. MDD has been associated with altered striatal function [54], and with reward processing [55]. Since reduced serotonergic pathway signalling in the striatum was recently associated with MDD [56], it suggests a mediating effect of PCLO in MDD. Also, these findings are consistent with a recent proposition that PCLO may be particularly relevant for reward processing deficits in the context of stress [57].

We found that processing of emotional words, irrespective of valence, was modulated by the *PCLO* risk allele in regions including insula, IFG, caudate head, and medial frontal gyrus, as shown when performing a conjunction analysis. Low caudate activity has previously been associated with both altered inhibition of negative stimuli in subjects at risk for MDD [58] and motivational pathway dysfunction, or the inability to experience pleasure or engage in rewarding activities [59]. We propose that lower insula, and IFG activity in PCLO+ carriers may reflect a general inadequacy for regulating emotional responsiveness, either to enhance or induce a positive emotional mood state, or to down regulate negative mood states, increasing vulnerability for MDD in PCLO+ carriers.

In the present study, no effect of genotype during successful recognition was found. However, we found a blunted activity in the amygdala in healthy PCLO+ carriers and in MDD patients irrespective of genotype during recognition of new positive words, relative to old words, which underlines that new positive information results in less salience signalling in the amygdala in *PCLO* risk allele-carriers. This neural variation in processing novel positive information may further contribute to development of MDD symptomatology, as novel positive information appears to go undetected and may therefore further contribute to a negative biased orientation towards the world. Nevertheless, results suggested that PCLO may play a modest role in biased processing of familiar information: in PCLO+ carriers, processing of negative familiar information was associated with insular hyper-activation, as well as hypo-activation in a network implicated in reward processing and learning (including the inferior and medial frontal, hippocampal, caudate head, insula, and putamen), compared to non-risk carriers. However, given that these latter results were subtreshold only, we may conclude that PCLO predominantly affects deep processing during of semantic classification and successful encoding of novel information, which is indirect supported by animal studies showing that novel information contributes to fear aspects of depressive-like behaviour (i.e. the depression phenotype) [60].

In the present study, similar results were obtained when repeating our main analyses after excluding SSRI users, which is in line with previous findings in an emotional face processing task [12]. The recent proposal that PCLO enhances the neurophysiologic response to SSRIs in MDD patients [16] is not supported by our study.

Some limitations should be noted. First, although we used similar 3T systems at each site in this multicenter study, no systematic scanning site × diagnosis bias occurred. However, variability in image acquisition may have occurred due to minor differences in hardware (receiver coil), imaging parameters, and timing of software upgrades. Second, depression severity in our MDD patients was only mild to moderate, due to recruitment from the general population, general practitioners, and outpatient mental health organizations, but not from inpatient clinics. Consequently, we do not know whether our interaction findings would have been more robust when inpatients had also been included. Third, cell sizes were small when testing for genotype × group interactions, which may have biased our results. To increase cell sizes in neuroimaging genetic studies using GWAS as genotypic factor, correction for multiple testing requires very large sample sizes (including healthy controls), which is only feasible in a multicenter meta-analysis approach [61]. However, the present study was a follow-up of a previous GWAS for the clinical phenotype of MDD, testing only a single promising polymorphism in the *PCLO* gene.

Although this study provides evidence for modulation of negative word encoding related activity by *PCLO* genotype, its role in the serotonergic pathway remains unclear and should be the focus of future research. A promising approach is likely to be the use of positron emission tomography (PET) tracers to study radioligand binding to receptors that interact with PCLO, as shown when studying genes associated with serotonin transporter function [62]. In addition, the use of longitudinal MRI designs may be helpful to investigate whether PCLO+ carriers continue to show a negative bias reflected in lower frontostriatal activity and therefore may, indeed, be more vulnerable to develop MDD.

### Conclusion

Our findings indicate that the presence of the *PCLO* risk allele may increase vulnerability for MDD by affecting the mood regulating capacity of the brain and by influencing dysfunctional reward processing in MDD. It further increases vulnerability for MDD by contributing to a general inadequacy for regulating emotional responsiveness. The interaction between the *PCLO* genotype and MDD reflected in decreased activity in the ventral striatum and amygdala also suggests that the pathophysiology of MDD is complex and may interact with the *PCLO* genotype. Moreover, we have found similar regions as MDD studies during emotional encoding, which indicates that the *PCLO* risk allele plays an important role in the mediation between MDD and altered brain activity.

## Supporting Information

File S1 Figure S1: Flowchart of participants included in this study. NESDA: Netherlands Study of Depression and Anxiety; PCLO+: *PCLO* risk allele carriers; PCLO−: *PCLO* non-risk allele carriers; n: number of participants. Table S2: Optimal threshold calculation for multiple comparison correction. Table S3: Main effect of encoding and recognition, specified per valence. Table S4: PCLO genotype effect during task. Figure S5: Parameter estimates of correctly recognized old versus new words. Effect is shown at the amygdala (MNI [−27 −3 −24]: during rejection of positive new words, healthy PCLO− carriers showed increased left amygdalar activation, while no difference between processing positive old and new words was observed in PCLO+ carriers and in MDD patients, indicating blunting to novel positive information in PCLO+ carriers and patients. PCLO: Piccolo genotype; PCLO+: PCLO risk allele carriers; PCLO−: PCLO non-risk allele carriers; MDD: Major Depressive Disorder; HC: Healthy controls; AU: arbitrary units.
(DOC)
