# Correction: Modulatory Effects of the Piccolo Genotype on Emotional Memory in Health and Depression

**DOI:** 10.1371/annotation/47489033-f246-4017-93d3-7bb9c43c796b

**Published:** 2013-08-21

**Authors:** Saskia Woudstra, Marie-José van Tol, Zoltán Bochdanovits, Nic J. van der Wee, Frans G. Zitman, Mark A. van Buchem, Esther M. Opmeer, André Aleman, Brenda W. Penninx, Dick J. Veltman, Witte J. Hoogendijk

There were multiple errors and an omission in the Funding Statement. The Funding Statement should read: "The infrastructure for the NESDA study (www.nesda.nl) is funded through the Geestkracht program of the Netherlands Organization for Health Research and Development (Zon-Mw, Grant number 10-000-1002) and is supported by participating universities and mental health care organizations (VU University Medical Center, GGZ inGeest, Arkin, Leiden University Medical Center, GGZ Rivierduinen, University Medical Center Groningen, Lentis, GGZ Friesland, GGZ Drenthe, Scientific Institute for Quality of Healthcare (IQ healthcare), Netherlands Institute for Health Services Research (NIVEL) and Netherlands Institute of Mental Health and Addiction (Trimbos Institute). This work was supported by the Top Institute Pharma, project number T5-203. The funders had no role in study design, data collection and analysis, decision to publish, or preparation of the manuscript." 

