# Supplementary material for: Modulatory Effects of the Piccolo Genotype on Emotional Memory in Health and Depression
Source: PLoS One. 2013 Apr 19;8(4):e61494. doi: 10.1371/journal.pone.0061494 (PMC3631241; doi:10.1371/journal.pone.0061494)
Supplement: File S1 — Figure S1: Flowchart of participants included in this study. NESDA: Netherlands Study of Depression and Anxiety; PCLO+: PCLO risk allele carriers; PCLO−: PCLO non-risk allele carriers; n: number of participants. Table S2: Optimal threshold calculation for multiple comparison correction. Table S3: Main effect of encoding and recognition, specified per valence. Table S4: PCLO genotype effect during task. Figure S5: Parameter estimates of correctly recognized old versus new words. Effect is shown at the amygdala (MNI [−27 −3 −24]: during rejection of positive new words, healthy PCLO− carriers showed increased left amygdalar activation, while no difference between processing positive old and new words was observed in PCLO+ carriers and in MDD patients, indicating blunting to novel positive information in PCLO+ carriers and patients. PCLO: Piccolo genotype; PCLO+: PCLO risk allele carriers; PCLO−: PCLO non-risk allele carriers; MDD: Major Depressive Disorder; HC: Healthy controls; AU: arbitrary units. (DOC) [file pone.0061494.s001.doc]

# Modulatory effects of the Piccolo genotype on emotional memory in health and depression

### Supporting information

**Figure S1: Flowchart of participants included in this study**


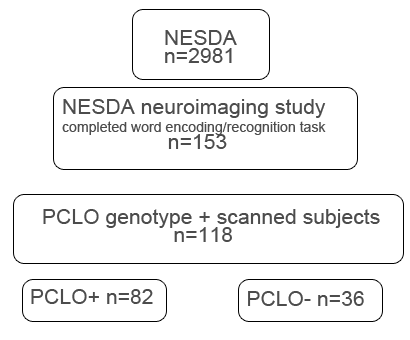


Flowchart of number of participants included in this study. n: number of participants; NESDA: Netherlands Study of Depression and Anxiety; PCLO: Piccolo genotype; PCLO+: *PCLO* risk allele carriers; PCLO-: *PCLO* non-risk allele carriers.

**Table** S2: Optimal threshold calculation for multiple comparison correction

|  | mean correlation | critical p-value |
| --- | --- | --- |
| Positive > neutral encoding | 0.63 | 0.026 |
| Negative > neutral encoding | 0.75 | 0.032 |
| Positive > neutral recognition | 0.84 | 0.037 |
| Negative > neutral recognition | 0.69 | 0.029 |

Analysed using www.quantitativeskills.com/sisa/calculations/bonfer.htm; number of tests: 6 (ACC, IFG, amygdala, hippocampus, insula, striatum), alpha: .05

**Table S3**: Main effect of encoding and recognition, specified per valence

| **Negative word encoding** | | | | | | | | | | | | | | | | |
| --- | --- | --- | --- | --- | --- | --- | --- | --- | --- | --- | --- | --- | --- | --- | --- | --- |
|  |  |  | | | MNI coordinates | | | | | |  |  | |  | | |
| Regions | Side | BA | | | x | y | | z | | | F | p FDR corrected | | | | k^a^ |
| Inferior frontal gyrus | R | 13 | | | 39 | 24 | | 9 | | | >8 | <.05 | | | | 40 |
|  | R | 45 | | | 51 | 27 | | 3 | | | >8 | <.05 | | | | 40 |
|  | R | 47 | | | 42 | 21 | | -15 | | | >8 | <.05 | | | | 7 |
|  | R | 9 | | | 48 | 6 | | 33 | | | >8 | <.05 | | | | 278 |
| Medial frontal gyrus | L | BA10 | | | -3 | 57 | | -3 | | | >8 | <.05 | | | | 9 |
|  | R | BA6 | | | 3 | 0 | | 57 | | | >8 | <.05 | | | | 51 |
|  | L | BA9 | | | -6 | 51 | | 27 | | | >8 | <.05 | | | | 208 |
| Middle frontal gyrus | R | BA10 | | | 36 | 48 | | 30 | | | >8 | <.05 | | | | 6 |
|  | L | BA10 | | | -33 | 45 | | 27 | | | >8 | <.05 | | | | 4 |
|  | L | BA46 | | | -39 | 30 | | 21 | | | >8 | <.05 | | | | 2 |
|  | L | BA6 | | | -30 | -6 | | 54 | | | >8 | <.05 | | | | 4415 |
|  | R | BA6 | | | 36 | -6 | | 57 | | | >8 | <.05 | | | | 278 |
|  | R | BA9 | | | 36 | 27 | | 39 | | | >8 | <.05 | | | | 2 |
| Superior frontal gyrus | L | 9 | | | -3 | 57 | | 33 | | | >8 | <.05 | | | | 208 |
|  | R | 10 | | | 21 | 60 | | 24 | | | >8 | <.05 | | | | 208 |
|  | L | 10 | | | -24 | 60 | | 21 | | | >8 | <.05 | | | | 2 |
| Anterior cingulate | L | 24 | | | 0 | 33 | | 0 | | | >8 | <.05 | | | | 1 |
|  | L | 32 | | | -6 | 27 | | 30 | | | >8 | <.05 | | | | 51 |
|  | R | 32 | | | 6 | 18 | | 33 | | | >8 | <.05 | | | | 51 |
|  | L | 32 | | | -3 | 15 | | 33 | | | >8 | <.05 | | | | 51 |
|  | L | 24 | | | -3 | -9 | | 48 | | | >8 | <.05 | | | | 51 |
| Posterior cingulate | L | 29 | | | -3 | -48 | | 6 | | | >8 | <.05 | | | | 31 |
| Amygdala | L |  | | | -21 | -6 | | -24 | | | >8 | <.05 | | | | 11 |
|  | R |  | | | 30 | -6 | | -12 | | | >8 | <.05 | | | | 48 |
| Inferior parietal | R | 40 | | | 63 | -39 | | 27 | | | >8 | <.05 | | | | 1 |
| Fusiform | R | 19 | | | 45 | -69 | | -15 | | | >8 | <.05 | | | | 65 |
|  | L | 37 | | | -42 | -66 | | -18 | | | >8 | <.05 | | | | 52 |
|  | L | 37 | | | -39 | -54 | | -12 | | | >8 | <.05 | | | | 52 |
| Insula | R | 13 | | | 39 | 0 | | -9 | | | >8 | <.05 | | | | 48 |
|  | R | 13 | | | 45 | -45 | | 24 | | | >8 | <.05 | | | | 2 |
| Middle temporal | L | 21 | | | -51 | 0 | | -24 | | | >8 | <.05 | | | | 4415 |
|  | R | 37 | | | 48 | -63 | | -6 | | | >8 | <.05 | | | | 65 |
| Superior temporal | R | 21 | | | 51 | -24 | | -6 | | | >8 | <.05 | | | | 111 |
|  | R | 22 | | | 57 | 9 | | -3 | | | >8 | <.05 | | | | 4 |
|  | R | 38 | | | 48 | 6 | | -27 | | | >8 | <.05 | | | | 9 |
|  | L | 38 | | | -33 | 0 | | -12 | | | >8 | <.05 | | | | 13 |
| Hippocampus | L |  | | | -33 | -27 | | -9 | | | >8 | <.05 | | | | 1 |
| **Positive word encoding** | | | | | | | | | | | | | | | | |
|  |  | |  | MNI coordinates | | | | | | |  |  | | |  | |
| Regions | Side | | BA | x | | y | | | z | | F | p FDR corrected | | | k^a^ | |
| Inferior frontal gyrus | R | | 45 | 54 | | 24 | | | 6 | | >8 | 0.032 | | | 2 | |
|  | L | | 47 | -48 | | 18 | | | -6 | | >8 | 0.024 | | | 13 | |
| Medial frontal gyrus | L | | 10 | -3 | | 57 | | | -3 | | >8 | 0.001 | | | 180 | |
|  | L | | 10 | -6 | | 60 | | | 6 | | >8 | 0.005 | | | 180 | |
|  | L | | 10 | -3 | | 60 | | | 27 | | >8 | 0.032 | | | 180 | |
|  | L | | 6 | -3 | | -6 | | | 57 | | >8 | 0.027 | | | 5 | |
| Middle frontal gyrus | L | | 6 | -45 | | 3 | | | 51 | | >8 | 0.024 | | | 1 | |
| Anterior cingulate | L | | 24 | -6 | | -24 | | | 39 | | >8 | 0.018 | | | 24 | |
| Inferior parietal lobule | L | | 40 | -54 | | -27 | | | 30 | | >8 | 0.011 | | | 15 | |
|  | L | | 40 | -42 | | -39 | | | 51 | | >8 | 0.032 | | | 2 | |
|  | R | | 40 | 45 | | -33 | | | 42 | | >8 | 0.05 | | | 1 | |
| Precentral gyrus | L | | 6 | -30 | | -18 | | | 57 | | >8 | 0.007 | | | 41 | |
|  | L | | 6 | -33 | | -6 | | | 39 | | >8 | 0.02 | | | 41 | |
| Postcentral gyrus | L | | 3 | -18 | | -30 | | | 51 | | >8 | 0.008 | | | 18 | |
|  | R | | 3 | 57 | | -18 | | | 30 | | >8 | 0.008 | | | 20 | |
| Precuneus | L | | 31 | -6 | | -54 | | | 30 | | >8 | 0.018 | | | 10 | |
| Caudate body | L | |  | -12 | | -3 | | | 15 | | >8 | 0.043 | | | 2 | |
| Caudate tail | R | |  | 36 | | -18 | | | -12 | | >8 | 0.01 | | | 13 | |
| Middle temporal gyrus | L | | 21 | -51 | | 3 | | | -24 | | >8 | 0.039 | | | 3 | |
|  | L | | 22 | -54 | | -45 | | | -3 | | >8 | 0.044 | | | 3 | |
|  | L | | 39 | -54 | | -63 | | | 6 | | >8 | 0.006 | | | 120 | |
| Superior temporal gyrus | L | | 13 | -48 | | -48 | | | 18 | | >8 | 0.001 | | | 120 | |
|  | L | | 21 | -51 | | -27 | | | -6 | | >8 | 0.041 | | | 2 | |
| **Negative word recognition** | | | | | | | | | | | | | | | | |
|  |  | |  | MNI coordinates | | | | | | |  | |  |  | | |
| Regions | Side | | BA | x | | | y | | | z | F | | p uncorrected | k^b^ | | |
| Precentral gyrus | R | | 6 | 57 | | | -12 | | | 39 | >8 | | <.001 | 2 | | |
| Parahippocampal gyrus | R | | 28 | 27 | | | -18 | | | -24 | >8 | | <.001 | 2 | | |
|  | R | | 34 | 18 | | | -12 | | | -18 | >8 | | 0.001 | 6 | | |
|  | R | | 36 | 27 | | | -33 | | | -21 | >8 | | <.001 | 8 | | |
|  | L | | 36 | -27 | | | -36 | | | -21 | >8 | | 0.001 | 1 | | |
| Hippocampus | L | |  | -33 | | | -15 | | | -24 | >8 | | 0.001 | 1 | | |
| Lingual gyrus | R | | 17 | 12 | | | -87 | | | -6 | >8 | | <.001 | 12 | | |
|  | L | | 17 | -9 | | | -87 | | | -3 | >8 | | 0.001 | 3 | | |
| Middle temporal gyrus |  | | 21 | 57 | | | -3 | | | -12 | >8 | | <.001 | 7 | | |
| Superior temporal gyrus |  | | 21 | 63 | | | -15 | | | -3 | >8 | | <.001 | 7 | | |

Main effect of encoding and recognition, specified per valence. MNI, Montreal Neurological Institute; BA, Brodmann Area; k, cluster size L, left; R, right.

a: clustersize at *p*<.05 FDR whole brain corrected

b: clustersize at *p*<.001 uncorrected

No main effect of positive word recognition was found

**Table S4**: PCLO genotype effect during task

| **Negative word encoding** | | | | | | | | | |
| --- | --- | --- | --- | --- | --- | --- | --- | --- | --- |
|  |  |  |  | MNI coordinates | | |  |  |  |
| Contrast | Regions | Side | BA | x | y | z | Z | p FDR^a^ | k^b^ |
| PCLO+ < PCLO- | ACC | L | 32 | -3 | 33 | 30 | 3.12 | 0.046 | 41 |
|  |  | R | 32 | 9 | 33 | 18 | 3.49 | 0.046 | 41 |
|  |  | L | 32 | -3 | 42 | -3 | 3.00 | 0.046 | 15 |
|  | IFG | L | 13 | -42 | 12 | 12 | 3.79 | 0.045 | 80 |
|  |  | L | 47 | -36 | 30 | -3 | 3.41 | 0.045 | 80 |
|  |  | R | 47 | 33 | 30 | -6 | 3.42 | 0.045 | 63 |
|  |  | L | 45 | -54 | 27 | 9 | 3.34 | 0.045 | 7 |
|  | Insula | R | N/A | 33 | 27 | -3 | 4.26 | 0.007* | 63 |
|  |  | R | N/A | 45 | 18 | -6 | 3.26 | 0.016* | 63 |
|  |  | L | 13 | -42 | 12 | 0 | 3.92 | 0.008* | 54 |
|  |  | R | N/A | 39 | -3 | 0 | 3.22 | 0.016* | 40 |
| **Positive word encoding** | | | | | | | | | |
|  |  |  |  |  |  |  |  | p uncorrected^a^ |  |
| PCLO+ < PCLO- | Inferior frontal gyrus | L | 47 | -45 | 15 | -6 | 3.26 | 0.001 | 3 |
|  | Precentral gyrus | R | 4 | 63 | -9 | 24 | 3.22 | 0.001 | 1 |
|  | Anterior cingulate | L | 24 | -3 | -9 | 24 | 3.2 | 0.001 | 4 |
|  | Hippocampus | R |  | 27 | -18 | -12 | 3.67 | <.001 | 2 |
|  | Caudate Head | R |  | 9 | 12 | -6 | 3.19 | 0.001 | 2 |
|  | Insula | L | 13 | -36 | 6 | 15 | 3.52 | <.001 | 2 |
|  |  | R | 13 | 45 | 3 | -9 | 3.94 | <.001 | 18 |
|  |  | R | 13 | 45 | -3 | 0 | 3.80 | <.001 | 9 |
|  |  | R | 47 | 33 | 18 | 0 | 3.80 | <.001 | 21 |
|  | Putamen | L |  | -15 | 15 | -6 | 3.59 | <.001 | 11 |
|  |  | L |  | -24 | 0 | -9 | 3.13 | 0.001 | 1 |
| **Negative word recognition** | | | | | | | | | |
|  |  |  |  |  |  |  |  | p uncorrected^a^ |  |
| PCLO+ > PCLO- | Paracentral lobule | L | 4 | -15 | -42 | 57 | 3.15 | <.001 | 1 |
|  | Precentral gyrus | R | 4 | 60 | -6 | 24 | 3.18 | <.001 | 3 |
|  | Anterior cingulate | L | 24 | -15 | 0 | 48 | 3.16 | <.001 | 1 |
|  |  | L | 24 | -18 | 3 | 51 | 3.11 | <.001 | 1 |
|  | Cuneus | L | 18 | -18 | -90 | 18 | 3.58 | <.001 | 7 |
|  | Lingual gyrus | R | 19 | 30 | -78 | -6 | 3.11 | <.001 | 1 |
|  | Inferior parietal lobule | R | 40 | 60 | -39 | 24 | 3.17 | <.001 | 1 |
|  | Precuneus | R | 7 | 12 | -45 | 54 | 3.33 | <.001 | 4 |
|  |  | R | 7 | 27 | -54 | 51 | 3.09 | <.001 | 1 |
|  | Supramarginal gyrus | L | 40 | -54 | -42 | 30 | 3.63 | <.001 | 3 |
|  | Insula | L | 13 | -30 | -27 | 21 | 3.45 | <.001 | 3 |
|  |  | R | 13 | 48 | -42 | 24 | 3.45 | <.001 | 6 |
|  | Middle temporal gyrus | R | 37 | 45 | -66 | 6 | 3.37 | <.001 | 6 |
|  |  | R | 39 | 45 | -69 | 21 | 3.35 | <.001 | 4 |
|  |  | R | 39 | 39 | -60 | 21 | 3.21 | <.001 | 1 |
|  | Superior temporal gyrus | R | 39 | 48 | -57 | 6 | 3.33 | <.001 | 1 |
|  |  | R | 39 | 48 | -54 | 15 | 3.2 | <.001 | 1 |
|  |  | L | 41 | -39 | -33 | 9 | 3.1 | <.001 | 2 |
|  |  | L | 42 | -60 | -27 | 12 | 3.23 | <.001 | 6 |

PCLO, piccolo genotype; PCLO+, *PCLO* risk allele carriers; PCLO-, *PCLO* non-risk allele carriers; MNI, Montreal Neurological Institute; BA, Brodmann Area; k, cluster size L, left; R, right.

a: PCLO genotype effects during positive word encoding, negative word recognition, and positive word recognition did not meet *P*<.05 FDR corrected. Therefore p-values of positive word encoding and negative word recognition are reported at *P*<.001 uncorrected. b: clustersize for the described threshold; * *P*<.05 FDR Bonferroni corrected [with a threshold of .032 for negative encoding]; No PCLO genotype effect during positive word recognition was found.

**Figure S5: Parameter estimates of correctly recognized old versus new words**

**
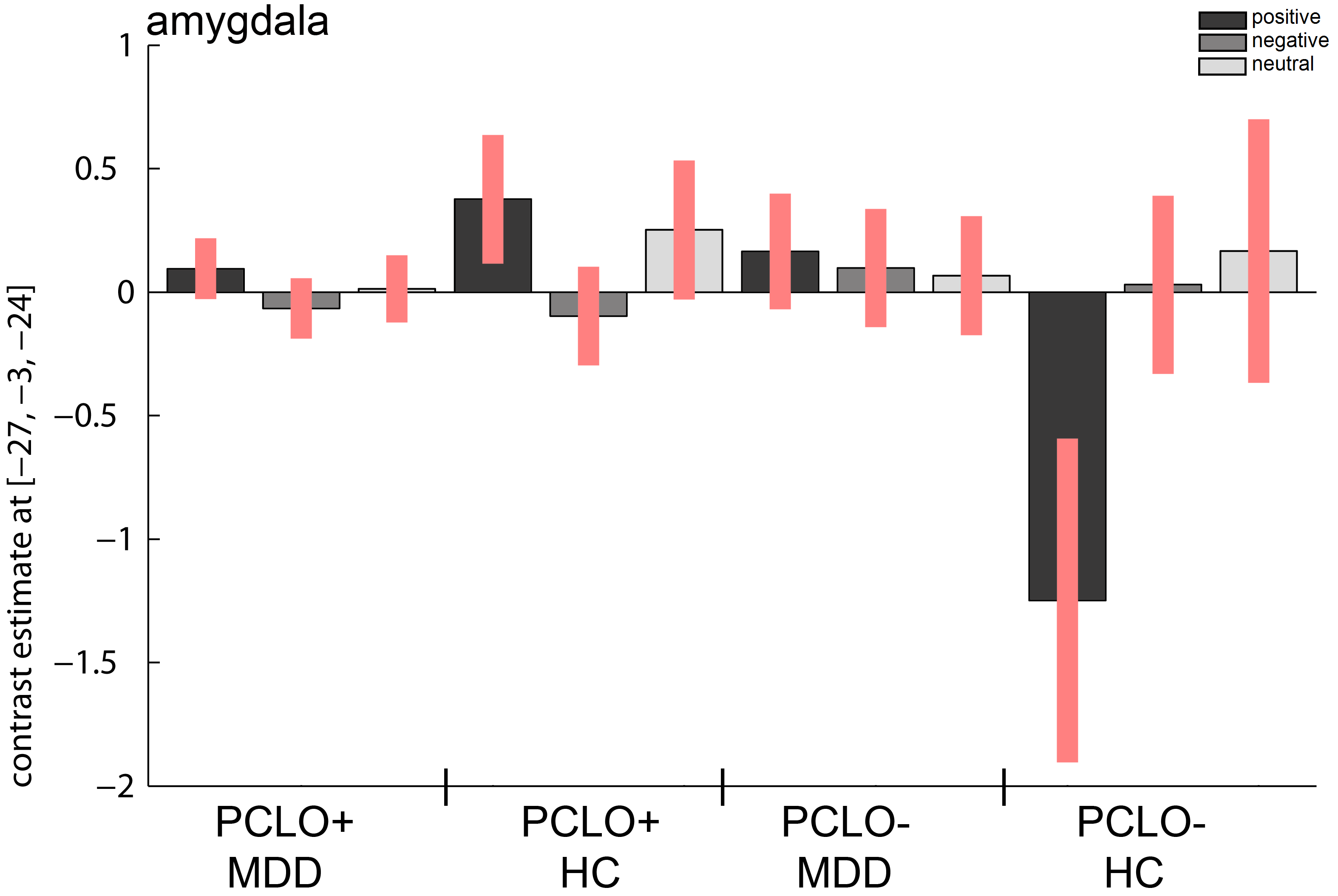
**
